# Research on Sleep Dynamics in Cleft Lip and Palate Patients Using Simple Sleep Testing

**DOI:** 10.3390/jcm12237254

**Published:** 2023-11-23

**Authors:** Naoko Nemoto, Hitoshi Kawanabe, Kazunori Fukui, Akihiko Oyama, Toru Okamoto, Kazuhiro Shimamura

**Affiliations:** 1 Department of Orthodontics and Dentofacial Orthopedics, Graduate School of Dentistry, Ohu University, Koriyama 963-8611, Japan; n-nemoto@den.ohu-u.ac.jp; 2 Division of Orthodontics and Dentofacial Orthopedics, Department of Oral Growth and Development, School of Dentistry, Ohu University, Koriyama 963-8611, Japan; k-fukui@den.ohu-u.ac.jp; 3 Department of Plastic and Reconstructive Surgery, Fukushima Medical University Hospital, Fukushima 960-1295, Japan; akihiko.oyama@mac.com; 4 Okamoto Orthodontic Clinic, Sapporo-shi 060-0001, Japan; info@siretukyosei.com; 5 Division of Pediatric Dentistry, Department of Oral Growth and Development, School of Dentistry, Ohu University, Koriyama 963-8611, Japan; k-shimamura@den.ohu-u.ac.jp

**Keywords:** cleft palate, apnea hypopnea index, out-of-center sleep testing (OCST), respiratory event index (REI)

## Abstract

Sleep-disordered breathing affects children’s growth and development, mental health, and learning ability. Postoperative scarring causes anteroposterior and vertical developmental disorders of the maxilla. Obstructive apnea is likely to occur due to the influence on the maxillofacial and airway morphology. In this study, we investigated the sleep-respiratory dynamics of school-aged children with unilateral cleft lip and palate by performing a simple overnight sleep study, maxillofacial morphology, airway analysis using lateral cranial radiographs, and model analysis. Children with unilateral cleft lip and palate showed a significantly higher respiratory event index (REI) than normal children; the maxilla was located in the posterior position in terms of maxillofacial morphology and airway morphology showed narrow values for all distance measurement items. Moreover, the width and length of the dental arch and the width of the alveolar base arch were significantly smaller. Furthermore, REI and SNA, ANB, and REI were negatively correlated with alveolar base arch width. Children with unilateral cleft lip and palate are more likely than normal children to develop sleep-disordered breathing due to increased airway resistance caused by undergrowth of the maxilla and narrowing of the upper airway and oral volume.

## 1. Introduction

Sleep-disordered breathing affects not only the growth and development of school-age children but also mental aspects, such as learning and depressive symptoms. Obstructive Sleep Apnea Syndrome (OSAS) is defined as “apnea that lasts for ≥10 s during bedtime, which occurs ≥ 30 times per night (7 h) or ≥5 times per hour” [1]. It causes a variety of disorders, such as daytime somnolence, fatigue, and a decline in learning ability due to reduced concentration [2]. The apnea–hypopnea index (AHI), which is essential for diagnosing OSAS, indicates the apnea–hypopnea index. Sleep apnea syndrome was considered when a patient experiences ≥ 5 apnea or hypopnea episodes per hour [3].

Out-of-center sleep testing (OCST) is a device that records movement during sleep and can be easily transported outside the laboratory to diagnose OSAS and assess therapeutic effects. The test estimates or supplements the results of the diagnosis, severity evaluation, and treatment effect evaluation by polysomnography [2]. Because the OCST does not record electroencephalograms, the total number of apnea and hypopnea events was calculated using the respiratory event index (REI) instead of the AHI [2].

Aside from obesity, male sex, and advanced age, OSAS is caused by a long soft palate, a low hyoid bone, receded mandible, and stenosis of the upper respiratory tract [3].

In this study, patients with cleft lip and palate who developed maxillary undergrowth as a result of lipoplasty or palatoplasty may have developed anteroposterior and vertical growth disorders, and upper airway stenosis may have caused sleep-breathing disorders compared to healthy children [4]. Postoperative maxillary growth has been a major problem since Grebern pointed out that palatoplasty using the Brophy method causes maldevelopment of the maxilla [3,5,6].

In patients with cleft palate, maxillary bone growth is inhibited by scar tissue after palatoplasty. It is hypothesized that the sleep-disordered breathing status of children with cleft palate is reduced compared with that of normal patients because the maxilla is highly associated with the nasal cavity and function. Therefore, the purpose of this study was to compare the effects of maxillary growth suppression on sleep-disordered breathing dynamics in patients with cleft palate, and thus on airway morphology, using a simple sleep test.

In this study, an OCST simplified overnight sleep study (Alice NightOne^®^ manufactured by Philips Respironics LLC, Murrysville, PA, USA) was performed on the sleep-breathing dynamics of school-age children with cleft lip and palate and compared with those of healthy children. Furthermore, the relationship between the upper respiratory area and oral cavity volume was examined for differences compared to healthy children.

## 2. Materials and Methods

The patients included ten patients who visited the orthodontic outpatient clinic at Ohu University Hospital and were diagnosed with unilateral cleft lip and palate (5 boys, 5 girls, average age 6.4 ± 0.9), as well as 13 healthy children with mild crowding who did not have syndromes or systemic diseases, had no nasopharyngeal disease, and had not undergone tonsillectomy (2 boys, 11 girls, average age 8.4 ± 1.3 years, below target group). The study schedule targeted patients who visited the hospital over a three-year period. The following items were investigated using the dental model, cephalometric standard radiograph, and simple overnight sleep study (Alice NightOne^®^ manufactured by Philips Respironics LLC, Murrysville, PA, USA) at the time of the first visit. The study was explained to the patients and their guardians. The results were used as research data only after obtaining informed consent. This study was approved by the Ethics Review Board of Ohu University (ethics review No. 328: approval date: 7 February 2021; facility number 11000803).

### 2.1. Simple Overnight Sleep Test

**Figure 1:**
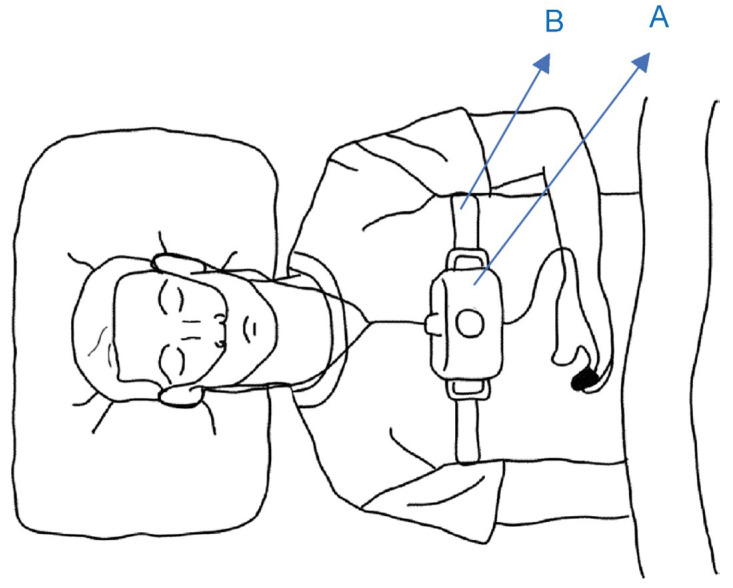
Attaching the Alice NightOne device. A: Alice NightOne unit; B: Constraining the respiratory belt.

**Figure 2:**
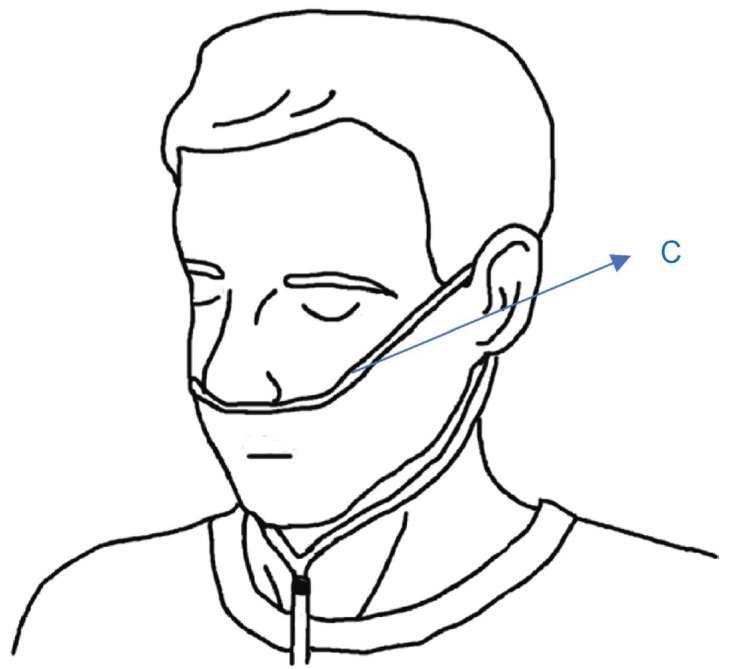
Nasal cannula attachment. C: Nasal cannula.

**Figure 3:**
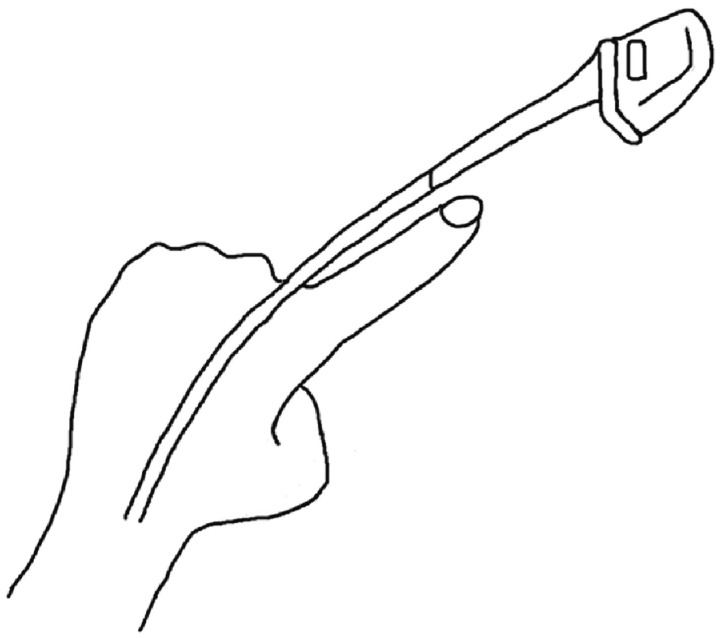
SpO_2_ sensor attachment. D:SpO_2_ sensor.

**Figure 4:**
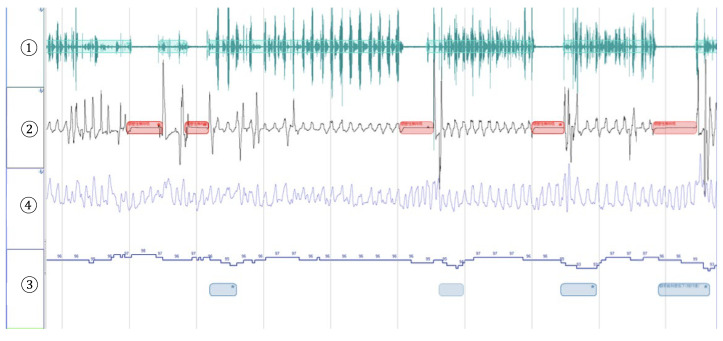
An example of analysis results. ① snore ② PFlow ③ THO ④SpO_2_ *: Oxygen desaturation (relative value).

**Figure 5:**
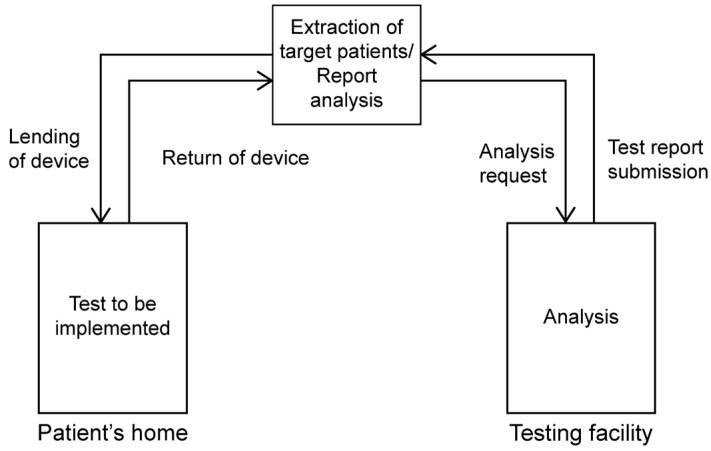
Analysis system.

The Alice NightOne device was placed on the solar plexus and secured using a constraining respiratory belt. A nasal cannula was attached to each patient. The SpO_2_ sensor included with the main unit was attached to the participant’s finger, and the child was instructed to go to bed (Figure 1, Figure 2 and Figure 3). Respiratory events were measured by chest movement from a constraining respiratory belt, respiratory events and snoring were measured by nasal pressure from a nasal cannula, and blood oxygen concentration and pulse were measured using an SpO_2_ sensor (Figure 4). The patient was instructed to use the product for two nights at home. The obstructive apnea index, central apnea index, mixed apnea index, and hypopnea index were measured using the International Classification of Sleep Disorders ICSD-2 as measurement items, and the REI was calculated. In addition, the recorded waveform complied with the American Academy of Sleep Medicine (AASM) Manual for the Scoring of Sleep and Associated Events ver.2.5, and the analysis was performed using 1A as the criterion for hypopnea. In addition, the REI was calculated using a Master Scorer (technician certified by the Japanese Society of Sleep Research) (Figure 5). In a report on accuracy control of sleep stage inspection judgment, the standard minimum match rate is ≥85% (K coefficient of 0.8 or more). Patients were instructed on how to attach the device themselves to avoid improper sensor attachment. If the recording time was short or an abnormality was detected in the sensor, we attempted to improve the accuracy and avoid any deficiencies in the recording by lending the device again and instructing the patient and guardians about the attachment method. According to the severity classification of AHI (number of apnea and hypopnea per hour of sleep), the severity of REI is classified into three categories: mild (5 to 15 times/h for mild), moderate (15 to 30 times/h), and severe (≥30 times/h). For statistical analysis, the Mann–Whitney U test and Spearman’s rank correlation coefficient were used. The significance level was set at *p* < 0.005.

### 2.2. Cephalometric Radiograph Image

#### 2.2.1. Cephalometric Analysis

Cephalometric radiographs were obtained according to the standard method, and the skeletal and dental patterns of the cleft lip, palate, and control groups were analyzed. Cephalometric radiographs of the facial skeleton were traced and analyzed.

**Figure 6:**
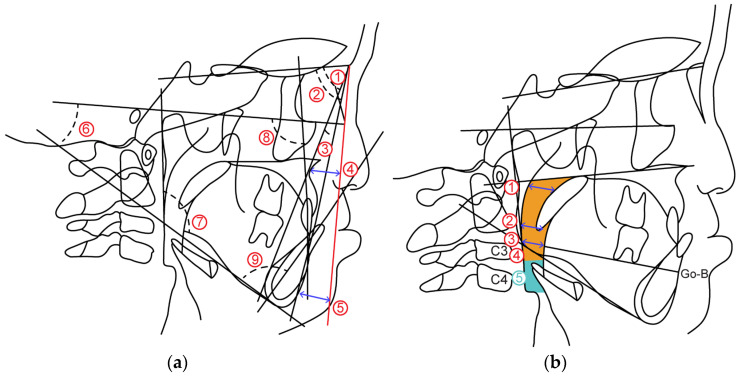
(**a**) Cephalometric analysis items by lateral cranial X-ray standard photograph. (**b**) Airway distance/area measurement point. Orange: Orpharynx; blue:Hypopharynx.

The measurement items are as follows. (Figure 6a).

(1) Skeletal pattern
  ① SNA: SN angle between plane and straight-line NA
  ② SNB: SN angle between plane and straight-line NB
  ③ ANB: Angle formed by straight line AN and straight line NB
  ④ McNamara to A: Distance from McNamara line to point A
  ⑤ McNamara to Pog: Distance from McNamara line to Pog
  ⑥ FH plane to Mandibular plane angle: Angle between mandibular plane and Frankfurt plane
  ⑦ Gonial Angle: Angle between mandibular plane and mandibular posterior marginal plane
(2) Dental pattern
  ⑧ U1 to FH plane angle: between maxillary central incisor tooth axis and Frankfurt plane Angle
  ⑨ L1 to Mandibular plane angle: Mandibular central incisor tooth axis and mandibular plane Angle

#### 2.2.2. Airway Distance, Area

Airway distance and area were measured based on the method of Alan A et al. [7] on the radiograph obtained from the lateral cephalometric radiograph (Figure 6b).

(1) Airway distance
  ① SPAS: Linear distance between the point where the axis parallel to the Go-B plane touches the posterior soft palate and the point where it touches the posterior pharyngeal wall.
  ② MAS: Linear distance from the point where a line parallel to the Go-B line passes through the lowest point of the soft palate and touches the posterior pharyngeal wall.
  ③ IAS: Linear distance between two points extending the Go-B line and intersecting the posterior border of the tongue and posterior wall of the pharynx.
(2) Airway area
  ④ Orpharynx: Area surrounded by a straight line connecting the point where the palatal plane intersects with the posterior pharyngeal wall, a straight line passing through the tip of the epiglottis parallel to the palatal plane and the posterior pharyngeal wall, hyoid bone, and soft palate wall.
  ⑤ Hypopharynx: Area bounded by two straight lines parallel to the Palatal plane passing through the hypopharyngeal area, tip of the epiglottis, anterior-inferior point of the fourth cervical vertebra, posterior pharyngeal wall, and posterior epiglottis wall.

### 2.3. Model Analysis

**Figure 7:**
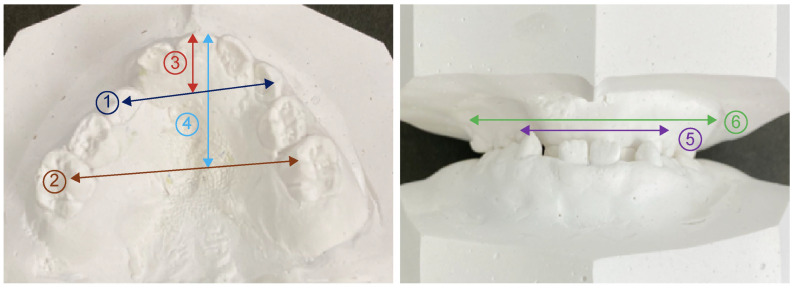
Model analysis items. ① Anterior width of maxillary coronal arch; ② Posterior width of maxillary coronal arch; ③ Anterior length of maxillary coronal arch; ④ Posterior length of maxillary coronal arch; ⑤ Anterior width of maxillary alveolar basal arch; ⑥ Posterior width of maxillary alveolar basal arch.

As the measurement points in the model analysis, the distance from the center of the line connecting the labial surfaces of both deciduous central incisors to the line connecting the cusps of both deciduous canines was defined as the anterior length of the maxillary coronal arch. The posterior length of the maxillary coronal arch was defined as the distance from the center of the line connecting the labial surfaces of both deciduous central incisors to the line connecting the most distal ends of the second deciduous molar on both sides. The distance between the deciduous canines and first deciduous teeth was defined as the anterior width of the maxillary coronal arch. The distance between the buccal grooves of the bilateral second deciduous molars was defined as the posterior width of the maxillary coronal arch. Regarding the width of the alveola basal arch, the distance between the deepest part of the gingiva, which corresponds to the apex of both primary canines and primary molars, is defined as the anterior width of maxillary alveolar basal arch. The distance between the deepest gingival regions corresponding to the apical regions of the second primary molars on both sides was defined as the posterior width of the maxillary alveolar basal arch (Figure 7).

## 3. Results

### 3.1. Simple Overnight Sleep Test

The REI values in the cleft lip and palate groups were significantly higher than those in the control group (*p* = 0.015). There was no significant difference in SpO_2_ between the two groups (Table 1).

### 3.2. Cephalometric Radiograph Image

#### 3.2.1. Cephalometric Analysis

In the skeletal system, the mean values of SNA and McNamara to A were lower in the cleft lip and palate group than in the control group, and there was a significant difference in SNA (*p* = 0.049). In the dental system, small and significant differences were observed between the U1 and FH and L1 and Mol groups (Table 2). We also found a negative correlation between the REI and SNA (*p* = 0.038), and a negative correlation between the REI and ANB, respectively (*p* = 0.028).

#### 3.2.2. Antero-Posterior Airway Distance and Area

SPAS, MAS, and IAS were significantly narrower in the cleft lip and palate groups than in the control group (*p* = 0.001), (*p* = 0.010), (*p* = 0.006), respectively. Both the oropharynx and hypopharynx showed small values, with the oropharynx showing a significant difference (*p* = 0.005) (Table 2).

### 3.3. Model Analysis

In the cleft lip and palate group, the anterior and posterior widths of the maxillary alveolar basal arch were significantly narrower than in the control group (*p* = 0.00). There was a narrow and significant difference in the anterior (*p* = 0.001) and posterior (*p* = 0.000). The length of the coronal arch was significantly shorter both anteriorly (*p* = 0.030) and posteriorly (*p* = 0.000) (Table 3). We also found a significant negative correlation between REI and the anterior width of the maxillary alveolar basal arch (*p* = 0.006).

## 4. Discussion

Cleft lip and palate is one of the most common oral diseases, varies by population, and its causes are highly dependent on genetic and environmental factors [8]. Evidence has implicated 40 genes involved, including 4p16(MSX1) and 1q32(IRF6) [9]. Patients with cleft lip and palate have speech, swallowing, and pronunciation problems; however, they also develop scar tissue after palatoplasty, inhibiting maxillary growth [4,10,11,12]. As such, early treatment and cooperation with various clinical departments are necessary, because this condition affects the maxillofacial morphology. In this study, we quantitatively evaluated sleep-breathing dynamics and the upper respiratory tract by comparing targeted patients with cleft lip and palate and healthy participants without cleft lip and palate. From sleep test results, first proposed by Guilleminault, OSA is a disease in which airway obstruction occurs during sleep, resulting in various symptoms, such as physical, mental, and learning aspects due to inadequate sleep [13]. Recently, a relationship has been identified between OSA and temporomandibular joint disorder and sleep bruxism (SB). If SB becomes habitual, it affects the bite, and over extended periods it can lead to temporomandibular joint disorder and muscle and skeletal disorders [14,15]. Additionally, poor sleep quality increases the incidence of dental caries [16] and sleep disorders and oral symptoms are closely related. Furthermore, in recent years, it has been suggested that the genetic code rs6313 HTR2A SNP is involved in the development of SB, and that the HTR2A rs2770304 polymorphism may affect the relationship between SB and OSA. It has also been shown that it is related to [17]. There are reports that SB caused by OSA is related to sleep habits and reduces sleep quality [18], so OSA affects not only academic and mental quality of life, but also oral health factors, such as temporomandibular joints, bite, and tooth decay. It is a serious disease that affects body-wide well being. PSG is considered the gold standard for the diagnosis of OSAS and one report evaluated the relationship between OSA and SB mentioned above from the first look at PSG [19]. However, it is difficult to perform PSG in all patients because of cost and patient burden. Although determining the sleep-breathing disorder index by a simple test is not currently defined as a diagnostic criterion, it is possible to judge the necessity of PSG for screening sleep-disordered breathing. Furthermore, the International Classification of Sleep Disorders (ICSD-3), issued in 2014, accepts the use of OCST for the diagnosis of OSAS in adults [20]. Murakami et al. [21] measured skeletal malocclusion using the simple sleep test equipment used in this study. Their results indicated that even a simple all-night sleep test can be evaluated as a sleep test. Therefore, we measured patients’ sleep dynamics using a simple overnight sleep test machine. In this study, although events in cleft lip and palate patients did not reach ≥ 5 times/hour, the REI value was significantly higher than that of the healthy participants. This may have been due to undergrowth of the maxillary bone caused by scarring from childhood palatoplasty, resulting in airway resistance and respiratory failure. Iwasaki et al. [22] reported that patients with cleft lip and palate had airflow obstructions in the nasal and nasopharyngeal cavities. Drettner et al. [23] reported deformation of the nasal septum, nasal obstruction, and thickening of the nasal mucosa in patients with cleft lip and palate. With these reports in mind, we believe that not only maxillary undergrowth, but also respiratory disorders are caused by higher nasal airway resistance in patients with cleft lip and palate compared to healthy participants.

Analysis of the maxillofacial morphology of patients with cleft lip and palate using cephalometric radiographs was reported by Graber in 1949 using lateral cranial radiographs [6,24]. However, SNA and SNB are facial skeletal morphologies that have a high risk of sleep-breathing disorders [25]. Soft tissue morphology findings indicate that children with cleft lip and palate are characterized by a small columellar angle, deviation of the base of the nasal alar and columella, and flattening of the nasal alar [26,27]. The results of this study also showed that the SNA, which indicates the anteroposterior positional relationship of the maxilla with respect to the cranium, was significantly smaller in patients with cleft lip and palate, based on lateral cranial radiographs. Furthermore, skeletal mandibular protrusion is related to the maxillary position and the upper airway narrows when the maxilla recedes, which means that moving the maxilla forward widens the airway area and improves sleep-breathing disorders [28]. This is thought to be due to anteroposterior growth and developmental disorders of the maxilla caused by palatoplasty performed in childhood [29]. 

In addition, SPAS, MAS, and IAS showed short airway distance measurements. Reiley et al. [30,31] stated that patients with OSA have a narrower SPAS than healthy participants. In this study, the SPAS also showed a significantly small and narrow value, indicating that stenosis of the SPAS is an influencing factor that causes respiratory changes. The upper airway cross-sectional area of patients with cleft lip and palate is approximately 70% that of healthy individuals. There are also reports that the upper airway is narrower in healthy participants [32,33]. In this study, the airway distance was significantly smaller for all measurement items, and the airway area was smaller in patients with cleft lip and palate, consistent with previous reports. In addition, the fact that the SNA was significantly smaller in the posterior position confirmed that the narrowing of the anteroposterior diameter of the upper airway was related to maxillary undergrowth.

The width of the coronal arch in a child with cleft lip and palate immediately after birth was larger than that in a healthy child. This implies lateral displacement of the maxillary segment and loss of continuity between the perioral muscles, such as the buccal and superior pharyngeal constrictor muscles and the muscles near the pharynx, which increase the width of the patient’s palate [34,35,36]. However, there was a marked reduction in the width after lip palatoplasty. In particular, the stenosis of the anterior part was remarkable, and the results of this study showed that the value of the maxillary dental arch was significantly smaller than that of healthy children. This is the effect of post-palatoplasty scar contraction due to the anterior extension of the rupture, and is consistent with the morphology of the maxillary arch in children with cleft lip and palate [37]. Moreover, in the width of the alveolar basal arch, Hama [38] reported developmental disorders of the maxillary alveolar basal as a characteristic of cleft lip and palate, and the anterior and posterior alveolar basal arch widths were narrow. In terms of the length of the coronal arch, children with cleft lip and palate showed smaller values for both the anterior and posterior lengths than healthy children. In addition, there was a negative correlation between REI and anterior alveolar basal arch width. In our study, cleft lip and palate had a high REI, and the model analysis showed significantly smaller values for the width of the coronal arch and alveolar basal arch, indicating stenosis. This is thought to be caused by postoperative scarring of the cleft lip and palate, resulting in undergrowth of the maxilla and narrowing of the intraoral volume, leading to an increase in airway resistance and REI.

### General Orthodontic Treatment for Children with Cleft Lip and Palate

Children with cleft lip and palate are prone to reverse skeletal occlusion due to maxillary cleft growth. In contrast, in orthodontic treatment during the growth period, a maxillary protractor (MPA) is widely used to improve jaw relationships by promoting the forward movement and growth of the jawbone. There are also reports of favorable treatment results due to forward growth of the maxilla using MPA [39,40,41,42,43,44]. In addition, as there are reports that moving the maxilla forward expands the upper airway and improves sleep-breathing disorders [28], the use of MPA in children with cleft lip and palate promotes anterior maxillary growth and increases the airway area, thus, affecting sleep respiratory dynamics. Therefore, further research on this topic is required.

## 5. Limitations

Several limitations of this study require mentioning. Under normal circumstances, research reliability must be improved by further increasing the number of participants in terms of clinical statistics. However, this study was conducted with children with cleft lip and palate, and among the patients who wished to receive orthodontic treatment at our hospital, which was inherently a limited population and could not be increased even if consent was obtained. Data collection was difficult and could not be performed if the patient was uncooperative or had a systemic disease.

## 6. Conclusions

In this study, we calculated the REI using a simple overnight sleep test for sleep-breathing dynamics in children with cleft lip and palate. Simultaneously, we analyzed skeletal morphology, airway distance, and airway area from lateral cranial radiographs. Furthermore, we compared the width and length of the coronal arches from the oral cavity model with those of healthy children and compared the characteristics of children with cleft lip and palate and the differences in respiratory disorders with those of healthy children. The results revealed that children with cleft lip and palate had a higher REI than healthy children, and their maxillofacial morphology showed that the maxilla was displaced posteriorly. Airway morphology was constricted based on the airway distance and area. In addition, the model analysis revealed that the anterior and posterior maxillary arch lengths were small, the anterior and posterior widths of the maxillary arch were narrow, and the anterior and posterior widths of the alveolar basal arch were narrow. A correlation was observed between the anterior width of the alveolar basal arch and REI. Based on these results, in children with cleft lip and palate, MPA is used to position the maxilla forward to improve postoperative maxillary tears, and use of an expansion bed to expand the intraoral volume without increasing airway resistance is important. Our future research goals include expanding upon the current findings with a larger sample of patients.

## Figures and Tables

**Table 1:** Simple overnight sleep test results. * *p* < 0.005.

	Cleft lip and Palate Group	Control Group	Significant Difference
REI	4.4 ± 2.9	1.8 ± 0.7	0.015 *
SP0_2_	96.3 ± 0.8	96.0 ± 0.6	0.446

**Table 2:** Cephalometric analysis results and Airway distance and area results. * *p* < 0.005; ** *p* < 0.001.

	Cleft lip and Palate Group	Control Group	Significant Difference
SNA	76.8 ± 4.2	80.2 ± 2.8	0.049 *
SNB	77.6 ± 2.7	77.6 ± 2.1	0.648
ANB	−0.7 ± 4.0	2.7 ± 1.9	0.003 **
McNamara to A	−2.2 ± 4.6	−1.3 ± 2.5	0.483
McNamara to Pog	−6.0 ± 4.8	−7.4 ± 3.8	0.483
FH plane to Mandibular plane angle	28.8 ± 4.8	30.5 ± 6.4	0.648
Gonial Angle	126 ± 7.1	127.0 ± 7.2	0.879
U1 to FH	93.6 ± 10.2	111.0 ± 5.2	0.000 **
L1 to Mandibular plane angle	80.1 ± 6.6	93.2 ± 8.7	0.000 **
APAS	7.8 ± 1.7	12.2 ± 3.4	0.011 *
MAS	8.2 ± 1.6	12.3 ± 3.4	0.010 **
IAS	9.5 ± 4.3	13.4 ± 3.7	0.006 **
Orpharynx	411.0 ± 105.3	564.6 ± 130.6	0.005 **
Hypopharynx	164.0 ± 44.2	195.7 ± 62.8	0.313

**Table 3:** Model analysis results. * *p* < 0.005; ** *p* < 0.001.

	Cleft lip and Palate Group	Control Group	Significant Difference
Anterior width of maxillary alveolar basal arch	36.4 ± 2.5	40.3 ± 2.6	0.000 **
Posterior width of maxillary alveolar basal arch	53.6 ± 3.8	59.4 ± 2.6	0.002 **
Anterior width of maxillary coronal arch	28.6 ± 2.3	33.0 ± 2.5	0.001 **
Posterior width of maxillary coronal arch	38.9 ± 3.2	47.2 ± 2.0	0.000 **
Anterior length of maxillary coronal arch	11.0 ± 1.7	12.9 ± 2.1	0.030 *
Posterior length of maxillary coronal arch	22.8 ± 3.6	32.4 ± 1.8	0.000 **

## Data Availability

Data are contained within the article. The data presented in this study are available in the Journal of Clinical Medicine.
